# Prognostic Nutritional Index as a Marker Associated With Disease Severity in Diabetic Kidney Disease: A Real-World North African Experience

**DOI:** 10.7759/cureus.114528

**Published:** 2026-08-14

**Authors:** Khawla Salhi, Salma Berghazi, Mohammed Amine Essafi, Zineb Elazime, Hayat Aynaou, Houda Salhi

**Affiliations:** 1 Department of Endocrinology, Diabetology, Metabolic Diseases and Nutrition, Hassan II University Hospital Center, Fez, MAR

**Keywords:** diabetes, diabetes research, diabetic kidney disease, immunonutritional status, prognostic nutritional index

## Abstract

Background: Diabetic kidney disease (DKD) is characterized by chronic inflammation and progressive protein-energy wasting. The Prognostic Nutritional Index (PNI) represents a promising marker for assessing immunonutritional deterioration. Our study aims to evaluate the PNI in patients with type 1 (T1D) and type 2 (T2D) diabetes mellitus with chronic kidney disease (CKD) across all its stages.

Materials and methods: A retrospective, cross-sectional analytical study was conducted among patients aged >18 years with T1DM and T2DM, followed between January 2025 and January 2026 at the Department of Endocrinology, Diabetology, and Metabolic Diseases, Hassan II University Hospital, Fez, Morocco. DKD was defined as an albumin-to-creatinine ratio (ACR) >30 mg/g and/or an estimated glomerular filtration rate (eGFR) <90 mL/min/1.73 m^2^ for ≥3 months. The PNI was calculated using the equation: $\mathrm{PNI} = \mathrm{Serum\ albumin}\ (\mathrm{g/L}) + 5 \times \mathrm{Lymphocyte\ count}\ (\times 10^{9}/\mathrm{L})$, with PNI ≥50 as normal nutritional status. We stratified patients into three groups: G1: ACR between 30 and 300 mg/g and/or eGFR between 60 and 90 mL/min/1.73 m^2^; G2: ACR >300 mg/g and/or eGFR <60 mL/min/1.73 m^2^ (moderate-to-advanced DKD); G3: eGFR <30 mL/min/1.73 m^2^ regardless of ACR (severe DKD). Exclusion criteria included incomplete medical records, active infection, cirrhosis, cancer, nephrotic syndrome, and chronic inflammatory conditions. Multivariate linear regression was adjusted for demographic factors, diabetes duration, and glycated hemoglobin (HbA1c). Statistical analyses were performed using IBM SPSS Statistics for Windows, Version 26 (Released 2018; IBM Corp., Armonk, New York, United States), with p < 0.05 considered statistically significant.

Results: Seventy-eight patients were included (41 T2DM and 37 T1DM). Mean age was 47.3 ± 11.4 years, with female predominance (male-to-female ratio: 0.41). Mean diabetes duration was 23 ± 7.3 years, and mean HbA1c was 11%. Mean PNI was 42.8 in G1 (n = 41), 35.6 in G2 (n = 21), and 28.1 in G3 (n = 16). Multivariate analysis showed a significant deterioration of immunonutritional markers with DKD progression (p < 0.05), with T1DM patients showing more marked kidney function worsening. PNI correlated positively with eGFR (p = 0.002) and inversely with ACR (p = 0.043). In linear regression, eGFR was independently associated with PNI (p = 0.002), and advanced nephropathy stage emerged as the strongest independent predictor of PNI decline (β = -6.47, 95% CI: -11.29 to -1.65, p = 0.011).

Conclusion: Immunonutritional status deteriorates progressively with the advancement of DKD. These findings suggest that PNI may represent a useful adjunctive marker of DKD to better identify patients at risk of protein-energy wasting and adverse outcomes.

## Introduction

Diabetic kidney disease (DKD) develops in approximately 30% of patients with type 1 diabetes (T1D) and 40% of patients with type 2 diabetes (T2D) and represents the leading cause of chronic kidney disease (CKD) and end-stage kidney disease worldwide [1]. According to the International Diabetes Federation (IDF) Diabetes Atlas, approximately 589 million adults aged 20-79 years were living with diabetes in 2024, a number projected to increase to 853 million by 2050 [2]. DKD progresses within a chronic inflammatory and catabolic environment whose nutritional repercussions are frequently overlooked.

Protein-energy wasting (PEW) is a well-known complication of CKD; it is defined as the loss of body protein and energy stores accompanying kidney disease [3]. In CKD, systemic inflammation reduces albumin synthesis and half-life, promotes muscle proteolysis and sarcopenia, and induces muscle insulin resistance, thereby making nutritional depletion possible even without reduced dietary intake [4]. In DKD, hyperglycemia-driven low-grade inflammation and oxidative stress lead to progressive renal injury and to a catabolic state, while high albuminuria adds a direct urinary loss of albumin. These processes affect the two laboratory parameters - serum albumin and the lymphocyte count - that constitute the Prognostic Nutritional Index (PNI) [5,6].

The PNI was initially introduced by Onodera et al. in 1984 as a perioperative nutritional risk score in malnourished patients undergoing gastrointestinal cancer surgery. It combines serum albumin and the total lymphocyte count into a single composite marker of immunonutritional status [7]. In comparison with other nutritional scores of DKD - the Geriatric Nutritional Risk Index (GNRI, originally established by Bouillanne et al., 2005) [8]), the Controlling Nutritional Status (CONUT, originally validated by Ignacio de Ulíbarri et al., 2005) [9]) score, and the Triglyceride-Total Cholesterol-Body Weight Index (TCBI; originally proposed by Doi et al., 2018) [10] - the PNI needs only two routinely available laboratory parameters and avoids the weight- and lipid-related factors that may be confounded by edema, fluid overload or dyslipidemia in diabetic patients [11]. This makes it particularly adapted to systematic monitoring in resource-limited settings. Nevertheless, data describing how the PNI behaves across the successive stages of DKD remain scarce, and no previous study has evaluated this behavior in a North African population.

We hypothesized that the PNI decreases progressively with worsening DKD severity and correlates positively with eGFR and inversely with ACR. This study aimed to evaluate the PNI in patients with diabetes mellitus across stages of DKD and to analyze its correlation with markers of kidney function.

## Materials and methods

This is a retrospective, cross-sectional analytical study conducted at the Department of Endocrinology, Diabetology, and Metabolic Diseases, Hassan II University Hospital, Fez, Morocco.

DKD was defined as an albumin-to-creatinine ratio (ACR) ≥30 mg/g and/or an estimated glomerular filtration rate (eGFR) <90 mL/min/1.73 m^2^, persisting for at least three months, in a patient with established diabetes mellitus [12].

Inclusion criteria

Patients aged >18 years with T1D and T2D diabetes mellitus who were followed between January 2025 and January 2026, agreed to participate in the study, and had complete medical records were included.

Exclusion criteria

Patients were excluded if they had incomplete medical records, refused to participate, had an active infection at the time of blood sampling, or had diagnosed chronic inflammatory conditions (cancer, cirrhosis, nephrotic syndrome, inflammatory bowel disease, rheumatoid arthritis, and similar), to avoid confounding of albumin and lymphocyte values.

Patient stratification

Patients were divided into three groups according to the severity of kidney involvement: G1: ACR between 30 and 300 mg/g and/or eGFR between 60 and 90 mL/min/1.73 m^2^ (early DKD); G2: ACR >300 mg/g and/or eGFR <60 mL/min/1.73 m^2^ (moderate-to-advanced DKD); G3: eGFR <30 mL/min/1.73 m^2^ regardless of ACR (severe DKD).

PNI calculation

The PNI was calculated from the same blood sample using the following validated formula: $\mathrm{PNI} = \mathrm{Serum\ albumin}\ (\mathrm{g/L}) + 5 \times \mathrm{Lymphocyte\ count}\ (\times 10^{9}/\mathrm{L})$. A PNI ≥50 was considered indicative of normal immunonutritional status.

Statistical analysis

Data were compiled using Microsoft Excel (Microsoft Corp., Redmond, WA, USA) and analyzed with IBM SPSS Statistics for Windows, Version 26 (Released 2018; IBM Corp., Armonk, New York, United States). Continuous variables are expressed as mean ± standard deviation, and categorical variables as percentages.

Multivariate linear regression adjusted for age, sex, diabetes duration, and glycated hemoglobin (HbA1c) was performed to evaluate the association between DKD stage and PNI. Pearson correlation was used to assess the relationship between PNI and kidney function parameters (ACR and eGFR). Diagnostic performance was evaluated using receiver operating characteristic (ROC) curve analysis, and the optimal clinical cut-off value was determined using the Youden Index [13]. Statistical significance was set at p < 0.05.

## Results

Patient characteristics

**Figure 1:**
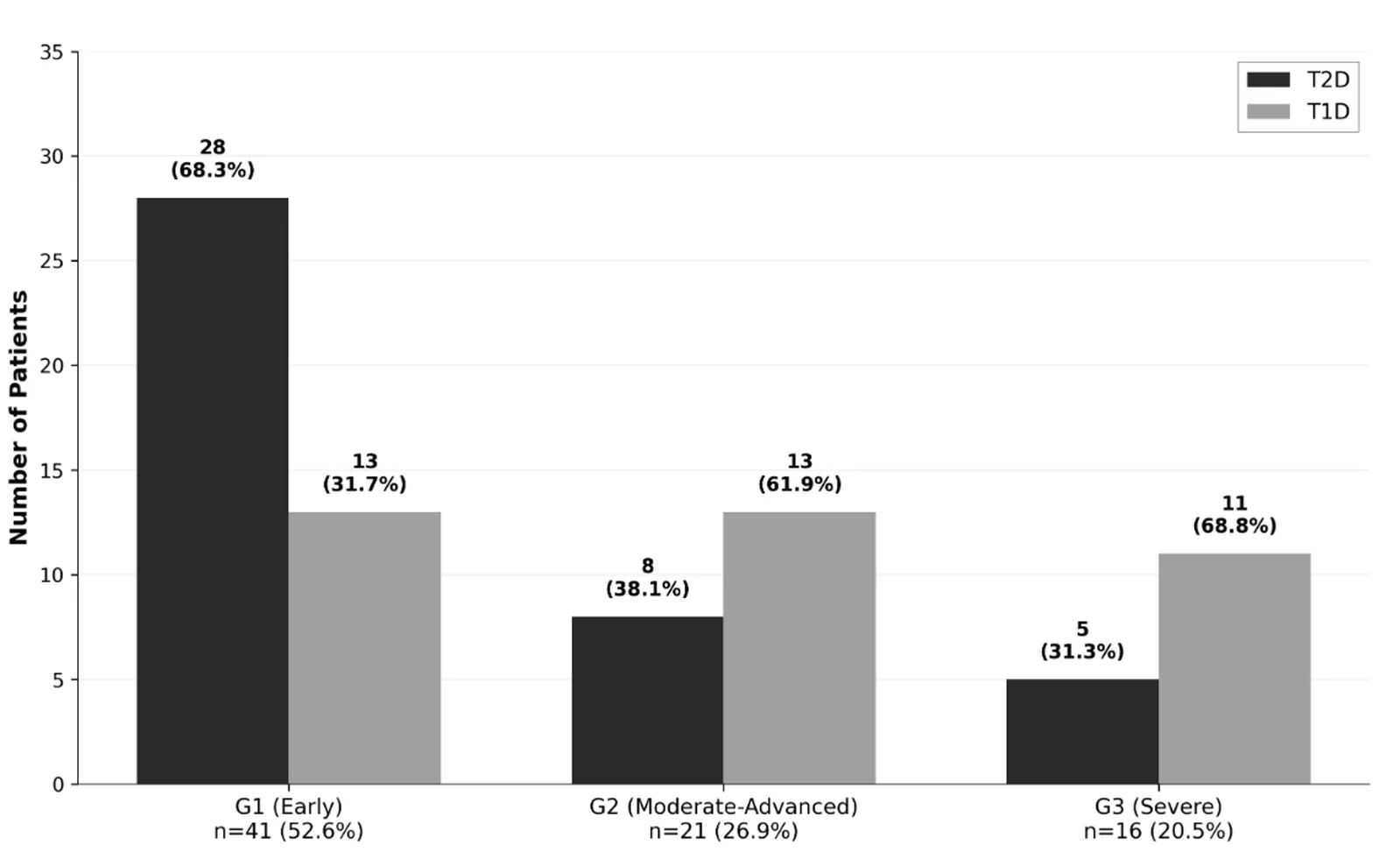
Distribution of type 2 diabetes mellitus (T2D) and type 1 diabetes mellitus (T1D) patients across diabetic kidney disease (DKD) severity groups. Data are presented as patient counts, n (%), within each group. The total cohort included 78 patients (100.0%): G1 (early DKD), n = 41 (52.6%), comprising 28 T2D patients (68.3%) and 13 T1D patients (31.7%); G2 (moderate-to-advanced DKD), n = 21 (26.9%), comprising 8 T2D patients (38.1%) and 13 T1D patients (61.9%); and G3 (severe DKD), n = 16 (20.5%), comprising 5 T2D patients (31.3%) and 11 T1D patients (68.8%).

A total of 78 (100%) patients with DKD were included: 41 (52.6%) with T2D and 37 (47.4%) with T1D. The mean age was 47.3 ± 11.4 years, with a female predominance (55 females (70.5%) vs. 23 males (29.5%); male-to-female ratio: 0.41). Mean diabetes duration was 23 ± 7.3 years. Mean BMI was 28.53 kg/m^2^, and mean HbA1c was 11 ± 0.5%. Patient distribution is illustrated in Figure 1.

PNI distribution across DKD stages

All 78 patients had a PNI below 50, confirming universal immunonutritional deterioration across the entire cohort. Group distributions and corresponding mean PNI values are summarized in Table 1.

**Table 1:** Baseline clinical, demographic, and immunonutritional characteristics stratified by diabetic kidney disease (DKD) severity groups. Categorical variables are represented as absolute frequencies and percentages (n (%)). Continuous variables with a normal distribution are represented as mean ± standard deviation (SD), whereas non-normally distributed continuous variables (urinary albumin-to-creatinine ratio) are represented as median values. Between-group comparisons between type 2 diabetes (n = 41) and type 1 diabetes (n = 37) were performed using two-tailed independent-samples Student's t-tests (t-statistic), Mann-Whitney U-tests (Z-statistic), or Pearson's chi-squared tests (χ^2^ statistic) as appropriate. Statistical significance was defined as p < 0.05, with p < 0.001 indicating high statistical significance. *Overall Pearson chi-squared test statistic (χ^2^ = 8.75, degrees of freedom df = 2, p = 0.013) assessing the proportion distribution of type 2 diabetes vs. type 1 diabetes across the three DKD severity strata.

Variable	Overall cohort (N = 78)	Type 2 diabetes (n = 41) (52.6%)	Type 1 diabetes (n = 37) (47.7%)	Test statistic and p-value
Demographic and clinical features				
Age (years), mean ± SD	47.3 ± 11.4	56.4 ± 8.2	37.2 ± 9.6	t = 9.52, p < 0.001
Sex (female), n (%)	55 (70.5)	29 (70.7)	26 (70.3)	χ^2^ = 0.002, p = 0.965
BMI (kg/m^2^), mean ± SD	28.53 ± 4.8	31.1 ± 3.9	25.7 ± 4.2	t = 5.89, p < 0.001
Diabetes duration (years), mean ± SD	23.0 ± 7.3	21.2 ± 6.4	25.0 ± 7.8	t = -2.36, p = 0.021
HbA1c (%), mean ± SD	11.0 ± 2.5	10.7 ± 2.1	11.3 ± 2.8	t = -1.08, p = 0.294
Renal function metrics				
eGFR (mL/min/1.73 m^2^), mean ± SD	77.2 ± 34.8	86.2 ± 28.4	67.2 ± 38.1	t = 2.51, p = 0.015
Urinary ACR (mg/g), median	56	42	85	Z = -2.51, p = 0.012
DKD severity distribution*, n (%)				
Group 1 (early DKD)	41 (52.6)	28 (68.3)	13 (35.1)	χ^2^ = 8.75, p = 0.013
Group 2 (moderate-to-advanced)	21 (26.9)	8 (19.5)	13 (35.1)	
Group 3 (severe DKD)	16 (20.5)	5 (12.2)	11 (29.7)	
Immunonutritional parameters				
Serum albumin (g/L), mean ± SD	33.9 ± 6.9	35.8 ± 4.9	31.8 ± 7.8	t = 2.74, p = 0.008
Total lymphocyte count (x10^9^/L), mean ± SD	1.2 ± 0.6	1.3 ± 0.5	1.1 ± 0.6	t = 1.60, p = 0.118
Prognostic Nutritional Index (PNI)				
Overall PNI score, mean ± SD	40.1 ± 8.0	42.8 ± 6.1	31.9 ± 8.4	t = 6.60, p < 0.001
PNI in Group 1 (early DKD), mean	42.8 ± 5.6	45.2	37.8	t = 2.47, p = 0.018
PNI in Group 2 (moderate-advanced), mean	35.6 ± 8.0	39.8	32.4	t = 2.23, p = 0.038
PNI in Group 3 (severe DKD), mean	28.1 ± 15.2	34.5	24.2	t = 3.33, p = 0.005

Immunonutritional status according to DKD severity

A total of 100% of our patients had a PNI score below the clinical normal threshold of 50, showing widespread immunonutritional deterioration. A decline in PNI score was observed along the progressive advancement of nephropathy; the mean PNI score fell from 42.8 ± 5.6 in the early-stage group (G1) to 35.6 ± 8.0 in the moderate-to-advanced group (G2) and reached its lowest point at 28.1 ± 15.2 in the severe kidney disease group (G3) (global p = 0.006).

Correlations between PNI and kidney function parameters

PNI and ACR

A significant inverse correlation was observed between PNI and ACR (Pearson r = -0.38, 95% CI: -0.56 to -0.17, p = 0.043; Spearman rho = -0.57, 95% CI: -0.70 to -0.40, p = 0.001), indicating that as ACR worsens, nutritional status deteriorates in parallel.

PNI and eGFR

**Figure 2:**
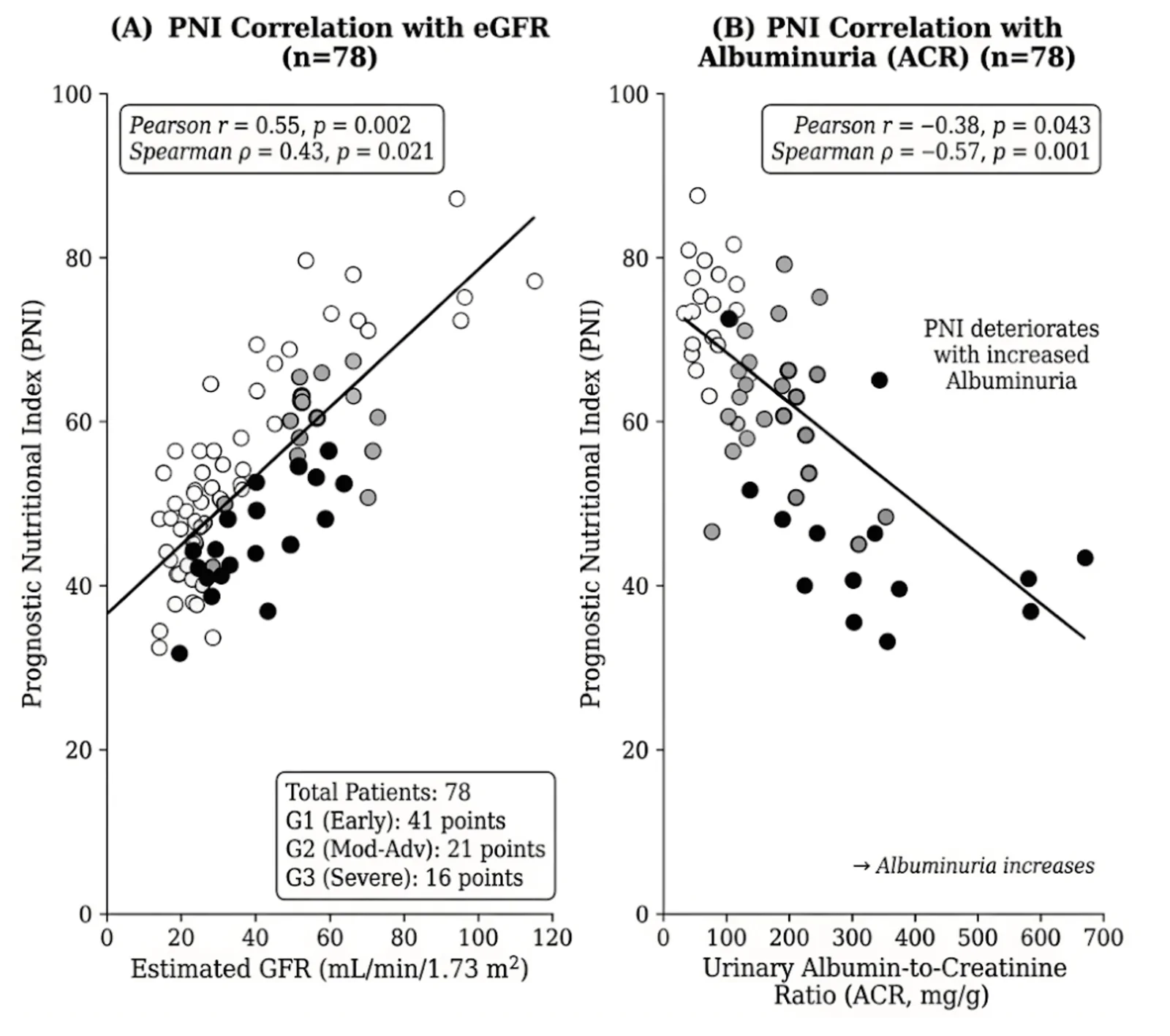
Correlation between the Prognostic Nutritional Index (PNI), estimated glomerular filtration rate (eGFR), and albumin-to-creatinine ratio (ACR). Correlations were assessed using Pearson’s correlation coefficient (r) and Spearman’s rank correlation coefficient (ρ), as reported in the Results. Statistical significance was set at p < 0.05.

A significant positive correlation was found between PNI and eGFR (Pearson r = 0.55, 95% CI: 0.37 to 0.69, p = 0.002; Spearman rho = 0.43, 95% CI: 0.23 to 0.60, p = 0.021), confirming that lower filtration rates are associated with lower immunonutritional reserve (Figure 2).

T1D vs. T2D comparison

**Figure 3:**
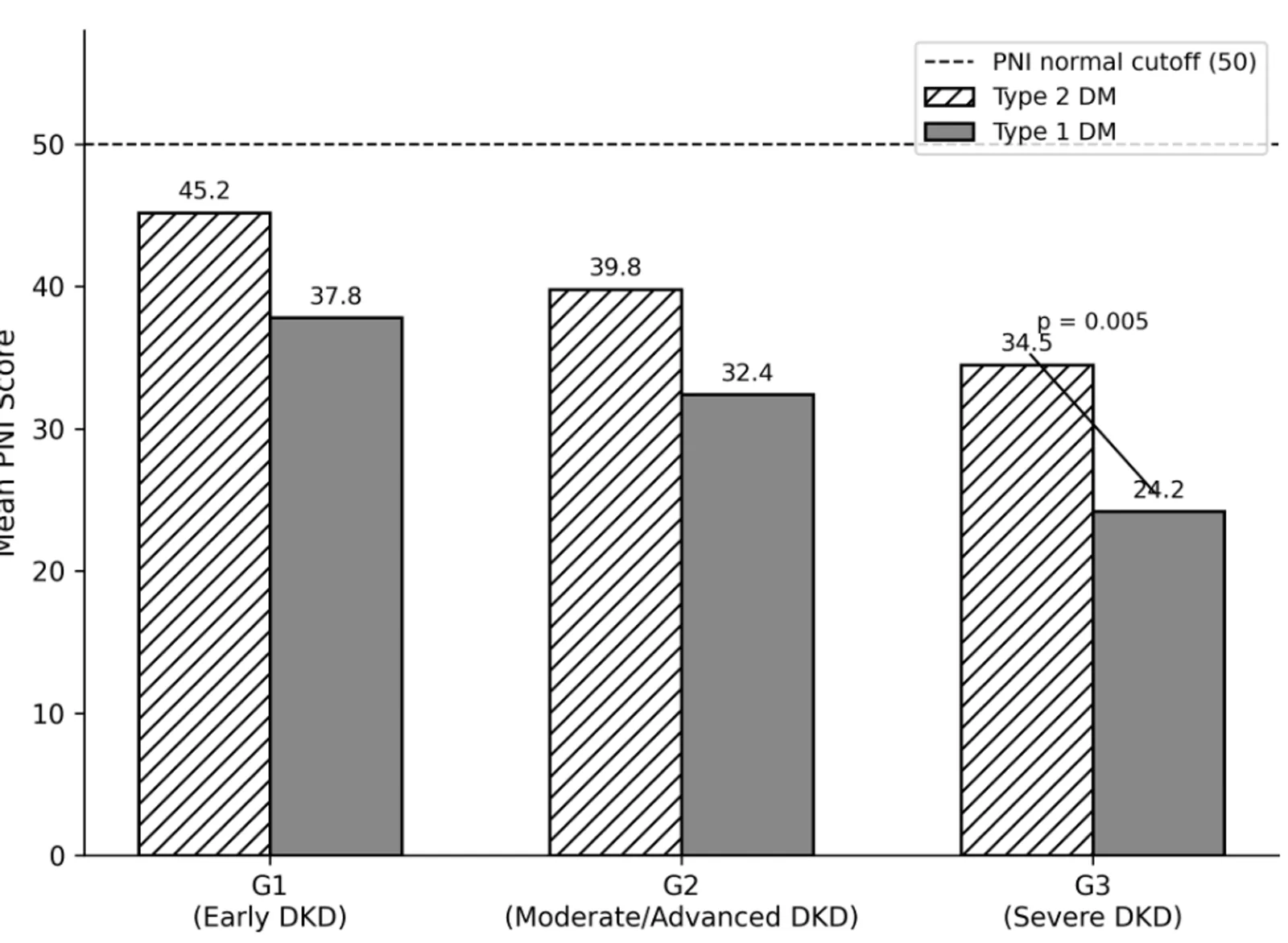
Distribution of the Prognostic Nutritional Index (PNI) between patients with type 1 diabetes mellitus (T1D) and type 2 diabetes mellitus (T2D). Data are presented as mean PNI values for each group (T2D, n = 41 (52.6%); T1D, n = 37 (47.7%)). p = 0.005. Statistical significance was set at p < 0.05. DKD: diabetic kidney disease

T1D patients showed consistently lower PNI values (p = 0.005, r = −0.38) at each DKD stage compared to T2D patients, with the clear difference observed in G3 (Figure 3).

Predictors of PNI deterioration

In the multivariate linear regression analysis, the PNI score was the continuous dependent variable. Adjusted for age, sex, diabetes duration, and overall metabolic control (HbA1c), the analysis revealed that an advanced DKD stage was a powerful independent predictor of PNI deterioration (β = -6.47, 95% CI: -11.29 to -1.65; p= 0.011). HbA1c (β = -0.34, 95% CI: -0.68 to -0.01; p = 0.044) and Diabetes duration (β = -0.18, 95% CI: -0.31 to -0.05; p = 0.006) also emerged as independent factors of nutritional decline. Demographic covariates, including age and biological sex, did not show independent statistical associations with PNI fluctuations (Table 2).

**Table 2:** Multivariable linear regression parameters detailing independent predictors of variation in the Prognostic Nutritional Index (PNI) score. A multivariable linear regression model was performed to evaluate independent clinical, metabolic, and demographic predictors of PNI score variation in patients with diabetic kidney disease (N = 78). PNI score was entered into the model as the continuous dependent outcome variable. Data are represented as unstandardized regression coefficients (beta), 95% confidence intervals (95% CIs), per-predictor Student's t-test statistics (t), and exact p-values. The multivariable regression model was simultaneously adjusted for baseline demographic covariates (age and biological sex), disease duration, and overall glycemic control (HbA1c). Statistical significance was defined at p < 0.05, with p < 0.001 denoting high statistical significance.

Predictor variable	Unstandardized coefficient (beta)	95% confidence interval (95% CI)	Test statistic (t)	p-value
Nephropathy stage	-6.47	-11.29 to -1.65	t = -2.63	0.011
Diabetes duration (years)	-0.18	-0.31 to -0.05	t = -2.71	0.006
HbA1c (%)	-0.34	-0.68 to -0.01	t = -1.99	0.044
Age (years)	-0.09	-0.41 to 0.24	t = -0.54	0.588
Sex	-2.39	-7.92 to 3.15	t = -0.85	0.383

**Figure 4:**
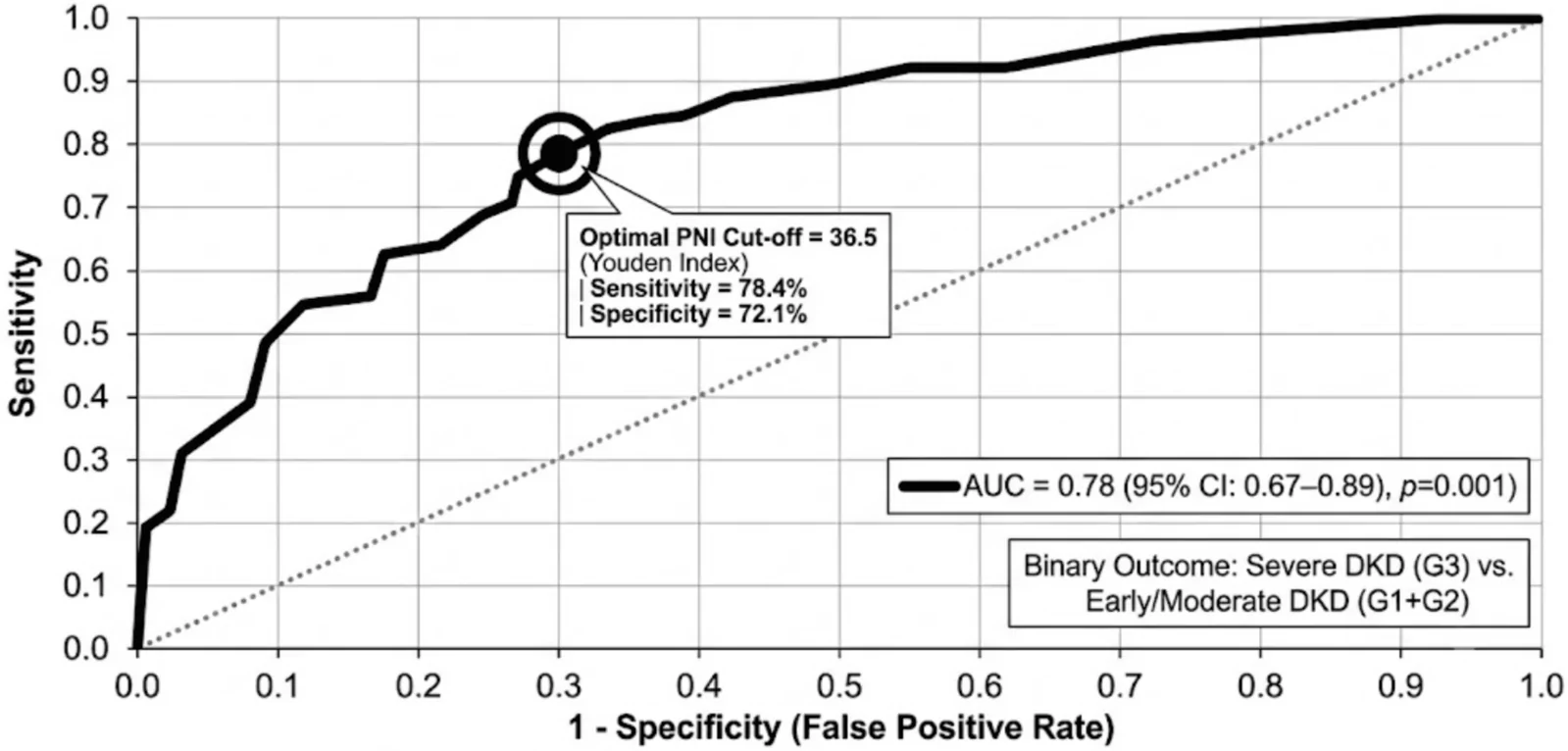
Receiver operating characteristic (ROC) curve analysis of the Prognostic Nutritional Index (PNI). AUC: area under the curve; DKD: diabetic kidney disease

To determine the optimal PNI threshold for identifying severe DKD, a ROC curve analysis (Figure 4) was performed by transforming the cohort into a binary outcome (severe DKD stage G3 = 1 vs. early/moderate stages G1+G2 = 0). The PNI showed a strong diagnostic performance in identifying severe disease, with an area under the curve (AUC) of 0.78 (95% CI: 0.67-0.89, p = 0.001). According to Youden index maximization, the optimal clinical cut-off value for PNI was identified at 36.5, yielding a sensitivity of 78.4% and a specificity of 72.1% for predicting severe nephropathy progression.

## Discussion

Our study highlights three findings: as a first result, the immunonutritional status, represented by the PNI (Onodera et al., 1984) [7], progressively deteriorates across the stages of DKD, falling from 42.8 ± 5.6 in early stage to 28.1 ± 15.2 in severe disease (p = 0.006). This decline was caused principally by the albumin factor: mean serum albumin fell stepwise across groups (36.1, 30.3 and 24.5 g/L; p = 0.015), whereas the lymphocyte count was decreasing slowly and non-significantly (1.3, 1.1 and 0.7 ×10^9^/L; p = 0.246), which is consistent with the inflammation-driven suppression of hepatic albumin synthesis and the urinary albumin loss of the advancing nephropathy [4-6].

Notably, 100% of our patients had a PNI score below the clinical normal threshold of 50, which means a widespread immunonutritional deterioration interpreted through three distinct lenses. First, the selection bias inherent to our tertiary hospital setting. Second, these cases were analyzed during a period of hospitalization, so they naturally represent a vulnerable and higher-risk subpopulation. Third, this raises an important question regarding whether a lower threshold should be established for metabolic kidney diseases. The standard PNI cut-off of 50 was originally proposed by Onodera et al. in 1984 to evaluate perioperative mortality risks in gastrointestinal surgical cancer patients [7]. In the context of a chronic, low-grade inflammatory state such as DKD, a threshold of 50 may be overly sensitive, thereby capturing early systemic and subclinical metabolic strain rather than traditional surgical malnutrition.

As a second result, the PNI is positively correlated with eGFR (r = 0.55, p = 0.002) and inversely with albuminuria (r = -0.38, p = 0.043), referring the nutritional deterioration directly to these two main markers, with nephropathy stage representing the strongest independent predictor of PNI on multivariate analysis (β = -6.47; p = 0.011). As a third result, the immunonutritional deterioration was more pronounced in patients with T1D than in those with T2D, indicating a vulnerability of that group.

These results are consistent with the large analysis of Xing et al. [11], as well as the cohort of Zhang et al., conducted in 14,349 participants with T2D, showing that a low PNI was independently associated with an increased risk of DKD and of all-cause mortality, which persisted after adjustment for metabolic and demographic factors and remained robust across subgroups [14]. Chronic hyperglycemia triggers the overactivation of the polyol and protein kinase C pathways, while accelerating the systemic accumulation of advanced glycation end-products (AGEs). This hostile environment promotes local renal and systemic oxidative stress and induces the release of key pro-inflammatory cytokines such as tumor necrosis factor-alpha (TNF-α) and interleukin-6 (IL-6). These mediators directly downregulate hepatic albumin gene expression and synthesis, while driving podocyte injury and endothelial dysfunction, leading to an aggravation of urinary protein loss and driving a destructive feed-forward loop between renal decline and nutritional wasting [15]. In comparison with our study, we found two main distinguishing matters: First, their cohort was cross-sectional and centred on the presence and prognosis of DKD, whereas we examined how the PNI behaves along the severity axis of the disease. Second, their analysis was confined to T2D, while we included both diabetes types. Our data extend Zhang et al.'s population-level risk association into a stage-dependent association within the DKD population.

Our findings also align with the cohort of Yu et al. (n = 1711), who showed that a low PNI - particularly when combined with elevated HbA1c - independently predicted early renal function decline [16]. They found an association between poor glycemic control and a low PNI, identifying the patients at greatest renal risk, which is directly relevant to our study, whose persistently high mean glycaemia is consistent with the inflammatory basis that underlies nutritional loss: hyperglycemia sustains low-grade inflammation, which suppresses albumin synthesis and promotes catabolism, lowering the PNI. This clinical synergy highlights a closed-loop metabolic-nutritional feedback system, wherein poorly managed hyperglycemia causes persistent tissue catabolism, while progressive renal impairment delays the clearance of circulating uremic toxins and inflammatory cytokines, which accelerates skeletal muscle wasting [16]. Whereas Yu et al. focused on the onset of renal decline in a preserved-function population, our study describes the same immunonutritional status across the full stages of DKD.

The marked impairment in our T1D patients may reflect several severe pathophysiological pathways. Beyond a longer cumulative lifetime exposure to hyperglycemia and chronic inflammation, T1D patients have insulinopenia. As insulin is the body’s principal anabolic hormone, its absolute absence severely cripples metabolic defenses against PEW, aggressively tilting the systemic balance toward muscle and protein catabolism [4]. Furthermore, it is characterized by major glycemic variability. These mechanisms promote severe oxidative stress and endothelial damage, generating a more aggressive inflammatory phenotype [17]. Additionally, it leads to more rapid and severe progression of albuminuria, resulting in massive urinary albumin leakage and depletion of circulating serum albumin stores [18].

Clinically, these results make the PNI a potentially useful marker for the nutritional follow-up of DKD. It requires only two routinely available laboratory parameters and avoids the weight- and lipid-related factors, which limit other indices, such as the GNRI and the CONUT score, in patients prone to fluid overload and dyslipidemia [8,19]. Its stage-dependent change may help detect the effect of disease progression as well as the effect of glycemic control on nutritional reserve. Incorporating this simple immunonutritional monitoring into routine DKD follow-up, alongside glycemic management, is in concordance with current Kidney Disease: Improving Global Outcomes (KDIGO) and Kidney Disease Outcomes Quality Initiative (KDOQI) nutrition recommendations [12,20].

For the strength of our study, it evaluated PNI across all DKD stages in a real-world North African cohort where earlier disease onset needs practical monitoring tools. The progressive decline in PNI from G1 to G3, coupled with its independent association with nephropathy stage, supports its integration into routine DKD follow-up. The inclusion of both T1D and T2D, combined with PNI's reliance on only two routine laboratory parameters, makes it particularly suited to resource-limited settings. However, the retrospective, cross-sectional design prevents assessment of PNI trajectories over time, limiting causal inference. The sample size (n = 78) from a single center restricts external validity, and our cohort's high mean HbA1c reflects persistently poor glycemic control that may limit generalization. Finally, the absence of complementary nutritional biomarkers alongside PNI limits contextualization within the broader nutritional landscape of DKD.

## Conclusions

Our study clearly demonstrates that immunonutritional status, as assessed by the PNI, deteriorates progressively and significantly with the advancement of DKD. All patients in our cohort had a PNI below the normal threshold of 50, underscoring the universal nutritional burden imposed by DKD regardless of stage. The significant correlations between PNI and both ACR and eGFR, along with the identification of nephropathy stage as the strongest independent predictor, position PNI as a potentially useful marker of DKD severity. The synergistic effect of poor glycemic control on PNI deterioration, alongside the more severe nutritional impairment observed in T1D patients, highlights the need for integrated glycemic and nutritional monitoring across all DKD stages. These findings support the routine integration of PNI into the clinical assessment of DKD to enable early identification of patients at risk of PEW and to guide timely nutritional intervention. Future research should focus on randomized clinical trials investigating whether optimizing immunonutritional status - thereby raising PNI scores - may help as a modifiable therapeutic target to delay the trajectory toward advanced CKD and end-stage renal disease.
